# Efficacy of galcanezumab in patients with migraine who did not benefit from commonly prescribed preventive treatments

**DOI:** 10.1186/s12883-021-02196-7

**Published:** 2021-04-23

**Authors:** Dulanji K. Kuruppu, Joshua Tobin, Yan Dong, Sheena K. Aurora, Laura Yunes-Medina, A. Laine Green

**Affiliations:** 1 Eli Lilly and Company, Indianapolis, IN USA; 2 Xenoscience, Phoenix, AZ USA; 3 Impel NeuroPharma, Seattle, WA USA; 4 Geisel School of Medicine at Dartmouth, Hanover, NH USA

**Keywords:** Galcanezumab, CGRP, Monoclonal antibody, Migraine, Fail preventive, Efficacy, Quality of life, CGRP mAb

## Abstract

**Background:**

Galcanezumab is a calcitonin gene-related peptide (CGRP) monoclonal antibody (mAb) indicated for the preventive treatment of migraine. While galcanezumab has demonstrated efficacy in patients who did not respond to prior preventive medications in general, its efficacy in patients who did not benefit from individual, commonly prescribed preventive treatments due to inadequate efficacy or safety/tolerability remains unknown.

**Methods:**

CONQUER was a 3-month, randomized, double-blind, placebo-controlled, phase 3b study that enrolled patients with episodic or chronic migraine who had 2 to 4 migraine preventive medication category failures in the past 10 years. Patients were randomly assigned 1:1 to receive placebo (*N* = 230) or galcanezumab 120 mg/month (240 mg loading dose; *N* = 232). Post hoc analyses were conducted to determine the efficacy of galcanezumab in patients who had not benefited from six of the most commonly prescribed migraine preventive medications. The mean change from baseline in monthly migraine headache days and ≥ 50 % response rates were assessed over months 1–3. Improvement in Migraine-Specific Questionnaire Role Function-Restrictive (MSQ-RFR) scores were assessed at month 3. The endpoints were estimated via mixed model with repeated measures.

**Results:**

The most common treatment failures due to inadequate efficacy or safety/tolerability, which at least 20 % of patients reported trying without benefit, included topiramate, amitriptyline, propranolol, valproate or divalproex, onabotulinum toxin A, and metoprolol. Patients who had not previously benefited from these treatments had a greater mean reduction in monthly migraine headache days across months 1–3 in the galcanezumab group compared to placebo (all *p* < 0.01). More patients treated with galcanezumab experienced a ≥ 50 % reduction from baseline in monthly migraine headache days across months 1–3 compared to placebo (all *p* < 0.05). Galcanezumab-treated patients had a greater improvement in mean MSQ-RFR scores at month 3 compared to placebo (all *p* < 0.01).

**Conclusions:**

In this population, galcanezumab was effective in reducing monthly migraine headache days, improving response rates, and enhancing quality of life in patients who had not previously benefited from topiramate, amitriptyline, propranolol, valproate or divalproex, onabotulinum toxin A, and/or metoprolol due to inadequate efficacy or safety/tolerability.

**Trial registration:**

ClinicalTrials.gov NCT03559257 (CONQUER).

## Introduction

Migraine is a chronic neurologic disease characterized by moderate-to-severe headaches with associated symptoms including nausea, vomiting, photophobia, or phonophobia that can be disabling [1]. Patients who experience four or more migraine headache days per month with some degree of impairment should be offered a migraine preventive medication [2]. While 39 % of patients with migraine qualify for prevention, only 13 % of patients currently use one [2]. The most commonly prescribed migraine preventive medication classes are antiepileptics (e.g., topiramate), beta blockers (e.g., propranolol), and tricyclic antidepressants (e.g., amitriptyline), none of which were specifically developed for the preventive treatment of migraine [3, 4]. Many oral preventive therapies require a slow titration schedule and more than one-half of patients who receive a prescription migraine preventive medication discontinue its use within 6 months of initiation [5–7]. Previous studies that assessed adherence to migraine standard-of-care treatments found that 6 months after treatment initiation with antidepressants, antiepileptics, or beta blockers, 68–73 % of patients were no longer taking these medications for migraine prevention [6].

In a cross-sectional study of US patients with migraine, 26.4 % of patients with episodic migraine (EM) and 53.3 % of patients with chronic migraine (CM) discontinued or switched their preventive treatment at least once. The main reasons for discontinuation/switching were inadequate efficacy and safety/tolerability [8]. Patients highlight efficacy and tolerability as the most important features of a preventive treatment; therefore, selecting a medication with these characteristics is crucial to ensuring optimal adherence [9, 10]. Persistence on migraine prophylaxis can, in turn, improve quality of life and patient outcomes [11–13].

Galcanezumab is a humanized monoclonal antibody (mAb) that selectively binds to calcitonin gene-related peptide (CGRP). The efficacy, safety, and tolerability of galcanezumab has been established for both EM and CM prevention [14–16]. However, these clinical trials excluded patients with a history of failure of three or more classes of migraine preventive treatments as defined by the American Academy of Neurology/American Headache Society treatment guidelines Level (A) and (B) evidence [17]. The CONQUER study demonstrated galcanezumab’s efficacy and safety in patients with EM and CM who had not benefited from 2 to 4 migraine preventive medication categories, with patients in the galcanezumab group experiencing 4.1 fewer monthly migraine headache days compared to 1.0 fewer in the placebo group from a baseline of 13.2 monthly migraine headache days [18]. This article assesses galcanezumab’s efficacy in the CONQUER study subpopulation who did not benefit from individual, commonly prescribed, migraine preventive medications.

## Methods

### Study design

This study includes post hoc analyses from the CONQUER trial that assessed galcanezumab’s efficacy in patients who had not benefited from 2 to 4 classes of migraine preventive treatments. A detailed description of the study design was reported previously [18]. Briefly, CONQUER (ClinicalTrials.gov registration number: NCT03559257; first posted date: 18/06/2018) was a phase 3b, multicenter (hospitals, clinics, or research centers in North America, Europe, and Asia), randomized, double-blind, placebo-controlled study. The trial included four study periods: (1) a 3–30 day screening period and washout of all preventive treatments; (2) a 1-month prospective baseline period; (3) a 3-month double-blind treatment period; and (4) a 3-month open-label treatment period. Participants were randomly assigned 1:1 to receive monthly subcutaneous injections of placebo or galcanezumab 120 mg following a loading dose of 240 mg. Randomization was done by a computer-generated random sequence and stratified by country and migraine frequency during the baseline period (low frequency EM, 4 to < 8 migraine headache days/month; high frequency EM, 8–14 migraine headache days/month and < 15 headache days/month; CM, ≥ 8 migraine headache days/month and ≥ 15 headache days/month). The study protocol was reviewed and approved by the appropriate institutional review board for each site and was conducted according to Good Clinical Practice and the Declaration of Helsinki guidelines. Patients provided written informed consent prior to initiating the study.

### Trial population

Participants were 18 to 75 years of age with a diagnosis of migraine as defined by International Classification of Headache Disorders – Third edition [1], a history of migraine for at least one year, and migraine onset prior to age 50. Eligible participants had to experience at least four migraine headache days and at least one headache-free day per month on average within the past 3 months. Patients were eligible if they had a history of documented failure of 2 to 4 migraine preventive medication categories in the past 10 years due to inadequate efficacy and/or tolerability. The medication categories included propranolol or metoprolol, topiramate, valproate or divalproex, amitriptyline, flunarizine, candesartan, botulinum toxin A or B (if taken for CM), and medications locally approved for prevention of migraine. Participants could continue the use of acute medications for migraine throughout the study. Patients with serious cardiovascular risk were not permitted to participate. The full list of inclusion and exclusion criteria was previously published [18].

### Outcomes measured

The post hoc analyses assessed change from baseline in mean monthly migraine headache days across months 1–3, proportion of patients achieving a ≥ 50 % response rate across months 1–3, and mean Migraine-Specific Questionnaire Role Function-Restrictive (MSQ-RFR) at month 3 for patients who did not benefit from individual, commonly prescribed, migraine preventive medications. The MSQ-RFR domain assesses migraine’s impact on patients’ ability to perform daily activities. The MSQ items are rated on a scale of 1 to 6 and then converted to a scale of 0 to 100, with higher scores indicating an improvement in functioning [19]. The individual preventive medications selected for this analysis were those that 20 % or more participants reported trying without adequate efficacy or tolerability. If patients did not benefit from or tolerate multiple medications, they could be assigned to more than one medication in this analysis. The efficacy of galcanezumab was further assessed for each prior migraine preventive based on failure due to (1) inadequate efficacy and (2) safety/tolerability.

### Statistical analysis

Analyses were performed in patients with an individual medication failure due to lack of efficacy or safety/tolerability in the total intent-to-treat population, which included all patients who were randomly assigned and received at least one dose of study drug. Mean change from baseline in number of monthly migraine headache days across months 1–3 and mean change in MSQ-RFR domain scores at month 3 were analyzed using a linear mixed model with repeated measures analysis. The model included treatment category, baseline migraine frequency category (low frequency EM, high frequency EM, and CM) [for MSQ-RFR outcome only], pooled country, month, and treatment-by-month interaction as well as baseline value and baseline value-by-month interaction. Baseline value referred to the baseline assessment for the analyzed outcome in the model. Proportion of patients achieving a ≥ 50 % response rate was assessed by a generalized linear mixed model with repeated measures. The model included fixed, categorical effects of treatment, month, and treatment-by-month interaction as well as baseline monthly migraine headache days. Unstructured covariance matrix was implemented in both linear and generalized linear mixed models to measure the correlation among repeated measures obtained on the same individuals. All statistical tests conducted were two-sided and *p*-values ≤ 0.05 were assumed to be statistically significant. No adjustments were made for multiple comparisons. Analyses were implemented using SAS Enterprise Guide 7.2 (SAS Institute, Cary, NC).

## Results

### Patient demographics and baseline disease characteristics

Patients were randomly assigned to placebo (*N* = 230) or galcanezumab 120 mg (*N* = 232). On average, patients were 46 years old, 86 % were female, and 82 % were Caucasian. Of the participants, 58 % had EM and 42 % had CM. The baseline number of monthly migraine headache days was 13.2, baseline MSQ-RFR score was 44.9, and patients averaged 3.5 preventive medication failures in their lifetime. These values were similar between treatment groups. The six most commonly failed migraine preventive medications in CONQUER, which at least 20 % of patients reported trying without adequate efficacy or tolerability, were topiramate, amitriptyline, propranolol, valproate or divalproex, onabotulinum toxin A, and metoprolol. The baseline monthly migraine headache days, MSQ-RFR scores, number of prior preventive medication failures, and the number of patients who did not benefit from treatment due to inadequate efficacy or safety/tolerability for each of these preventive medications are presented in Table 1.

**Table 1:** Baseline disease characteristics by previous preventive medication treatment failure^a^

Preventive medication	Number of monthly migraine headache days, mean (SD)		MSQ-RFR score, mean (SD)		Number of prior migraine preventive medications failed in lifetime, mean (SD)		Number of patients who did not benefit from treatment, n (%)	
	**PBO**	**GMB 120 mg**	**PBO**	**GMB 120 mg**	**PBO**	**GMB 120 mg**	**due to inadequate efficacy**^**b**^	**due to safety/tolerability**
**Topiramate**	13.1 (5.8)	13.5 (6.1)	42.4 (16.7)	43.9 (15.6)	3.6 (1.8)	3.7 (1.8)	270 (58.4)	95 (20.6)
**Amitriptyline**	13.4 (5.8)	14.3 (6.2)	43.8 (18.8)	45.5 (17.5)	3.7 (1.9)	3.9 (1.8)	215 (46.5)	69 (14.9)
**Propranolol**	14.4 (6.3)	14.4 (5.9)	44.3 (19.0)	44.0 (17.8)	4.3 (2.0)	4.1 (2.0)	135 (29.2)	26 (5.6)
**Valproate or divalproex**	12.6 (5.8)	13.2 (5.6)	46.7 (19.0)	49.1 (16.9)	3.8 (1.8)	3.8 (1.9)	123 (26.6)	39 (8.4)
**Onabotulinum toxin A**	15.9 (5.4)	16.5 (5.6)	40.3 (19.2)	42.2 (15.5)	4.8 (2.5)	4.3 (2.0)	94 (20.3)	1 (0.2)
**Metoprolol**	12.7 (5.3)	12.2 (5.8)	40.4 (18.0)	43.8 (14.9)	3.5 (1.5)	3.9 (1.8)	82 (17.7)	18 (3.9)

Abbreviations: *GMB* galcanezumab, *MSQ-RFR* Migraine-Specific Questionnaire Role Function-Restrictive domain, *n* number of patients within each specific category, *PBO* placebo, *SD* standard deviation

^a^Based on patients who did not benefit from treatment due to inadequate efficacy or safety/tolerability

^b^Includes inadequate response and/or no response

### Reduction in monthly migraine headache days

Patients who had not previously benefited from the six most commonly failed preventive medications in CONQUER (topiramate, amitriptyline, propranolol, valproate or divalproex, onabotulinum toxin A, or metoprolol) and were assigned to galcanezumab had a greater mean reduction in monthly migraine headache days across months 1–3 compared to placebo (all *p* < 0.01; Table 2). The mean change difference between galcanezumab and placebo was at least 2.5 monthly migraine headache days, regardless of the previously failed migraine preventive medication. The largest mean change difference between galcanezumab and placebo was seen in patients previously treated with onabotulinum toxin A (-6.3 monthly migraine headache days in the galcanezumab group vs. -1.5 in placebo).

**Table 2:** Mean reduction in monthly migraine headache days, 50 % response rates, and improvement in MSQ-RFR scores among patients who did not benefit from individual preventive medications

Preventive medication	PBO	GMB 120 mg	*p* value
**Topiramate**			
LS mean change from baseline in monthly migraine headache days across months 1–3 (SE)	-1.0 (0.4)	-4.2 (0.4)	< 0.0001
≥ 50 % reduction in monthly migraine headache days across months 1–3, % (SE)	14.7 (2.2)	37.8 (3.0)	< 0.0001
LS mean change from baseline in MSQ-RFR at month 3 (SE)	+ 10.4 (1.9)	+ 22.5 (1.9)	< 0.0001
**Amitriptyline**			
LS mean change from baseline in monthly migraine headache days across months 1–3 (SE)	-1.2 (0.5)	-4.3 (0.5)	< 0.0001
≥ 50 % reduction in monthly migraine headache days across months 1–3, % (SE)	13.9 (2.4)	37.4 (3.3)	< 0.0001
LS mean change from baseline in MSQ-RFR at month 3 (SE)	+ 10.4 (2.0)	+ 23.6 (2.1)	< 0.0001
**Propranolol**			
LS mean change from baseline in monthly migraine headache days across months 1–3 (SE)	-1.7 (0.6)	-4.3 (0.6)	0.0005
≥ 50 % reduction in monthly migraine headache days across months 1–3, % (SE)	14.9 (3.3)	34.5 (4.3)	0.0008
LS mean change from baseline in MSQ-RFR at month 3 (SE)	+ 10.4 (2.8)	+ 22.9 (2.7)	0.0001
**Valproate or divalproex**			
LS mean change from baseline in monthly migraine headache days across months 1–3 (SE)	-0.4 (0.5)	-3.6 (0.5)	< 0.0001
≥ 50 % reduction in monthly migraine headache days across months 1–3, % (SE)	10.6 (3.1)	33.8 (4.7)	0.0002
LS mean change from baseline in MSQ-RFR at month 3 (SE)	+ 6.1 (2.2)	+ 17.0 (2.3)	< 0.0001
**Onabotulinum toxin A**			
LS mean change from baseline in monthly migraine headache days across months 1–3 (SE)	-1.5 (1.0)	-6.3 (0.9)	< 0.0001
≥ 50 % reduction in monthly migraine headache days across months 1–3, % (SE)	9.9 (3.9)	38.7 (5.7)	0.0008
LS mean change from baseline in MSQ-RFR at month 3 (SE)	+ 5.4 (5.4)	+ 24.0 (5.2)	< 0.0001
**Metoprolol**			
LS mean change from baseline in monthly migraine headache days across months 1–3 (SE)	-0.3 (0.9)	-3.0 (0.9)	0.0023
≥ 50 % reduction in monthly migraine headache days across months 1–3, % (SE)	14.7 (4.1)	33.1 (5.4)	0.0112
LS mean change from baseline in MSQ-RFR at month 3 (SE)	+ 5.8 (3.4)	+ 16.5 (3.4)	0.0025

Abbreviations: *GMB* galcanezumab, *LS* least-squares, *MSQ-RFR* Migraine-Specific Questionnaire Role Function-Restrictive domain, *PBO* placebo, *SE* standard error

### 50% response rates

A greater proportion of patients treated with galcanezumab who previously did not benefit from the six most commonly failed preventive medications in CONQUER experienced a ≥ 50 % reduction relative to baseline in monthly migraine headache days across months 1–3 compared to placebo (all *p* < 0.05; Table 2). Patients who did not benefit from topiramate, amitriptyline, propranolol, or metoprolol were over two times more likely to achieve a ≥ 50 % response on galcanezumab compared to placebo. Patients who did not benefit from valproate/divalproex or onabotulinum toxin A were over three times more likely to achieve a ≥ 50 % response on galcanezumab compared to placebo.

### MSQ-RFR domain scores

There was a significant improvement in quality of life as measured by MSQ-RFR at month 3 in galcanezumab-treated patients who previously had not benefited from the six most commonly failed preventive medications in CONQUER compared to placebo (all *p* < 0.01; Table 2). For each of these prior preventive medications, patients who received galcanezumab had at least a 10-point greater increase in mean MSQ-RFR scores compared to patients who received placebo.

### Reduction in monthly migraine headache days by failure due to inadequate efficacy and safety/tolerability

Treatment failure was more often due to inadequate efficacy than safety/tolerability, regardless of prior preventive (Table 1). The most commonly failed preventive medications due to inadequate efficacy were topiramate (58.4 %), amitriptyline (46.5 %), and propranolol (29.2 %), while the most commonly failed preventive medications due to safety/tolerability were topiramate (20.6 %), amitriptyline (14.9 %), and valproate or divalproex (8.4 %) (Table 1).

**Fig. 1:**
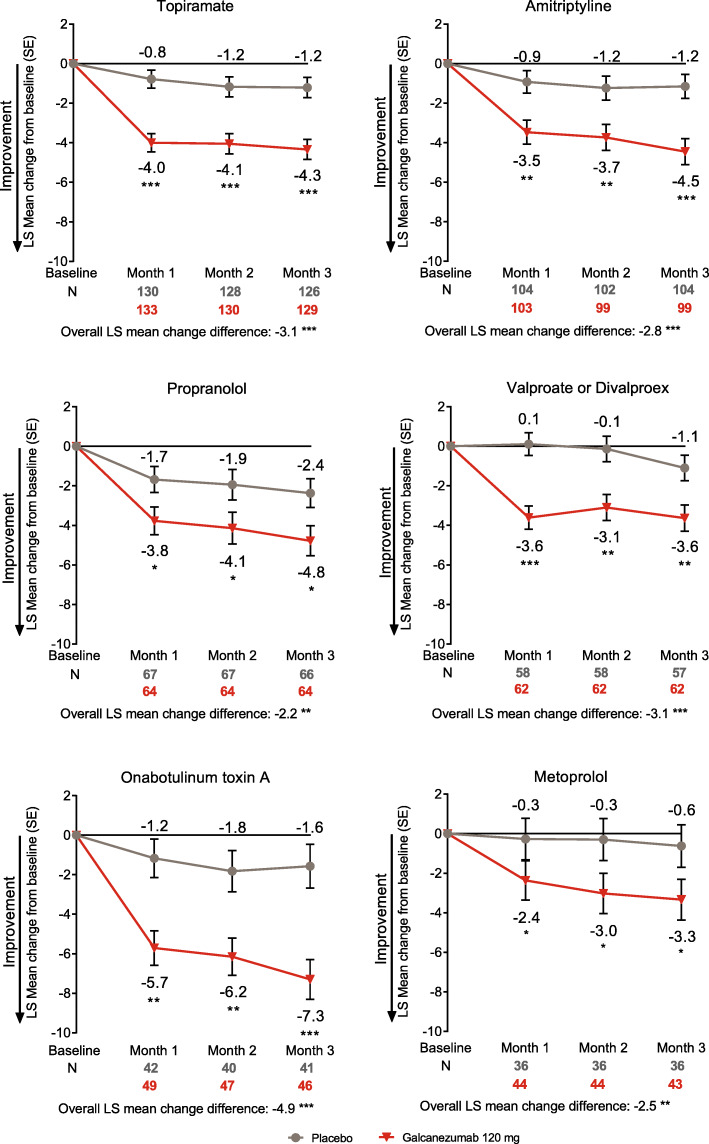
LS mean change from baseline in the number of monthly migraine headache days in patients who did not benefit from migraine preventive medication due to inadequate efficacy. *p<0.05, **p<0.01, ***p<0.0001 vs placebo. Abbreviations: *LS* least-squares, *N* number of intent-to-treat patients, *SE* standard error

Galcanezumab-treated patients who experienced inadequate efficacy with topiramate, amitriptyline, propranolol, valproate or divalproex, onabotulinum toxin A, or metoprolol all had a significant reduction in overall mean monthly migraine headache days across months 1–3 compared to placebo (all *p* < 0.01; Fig. 1).

**Fig. 2:**
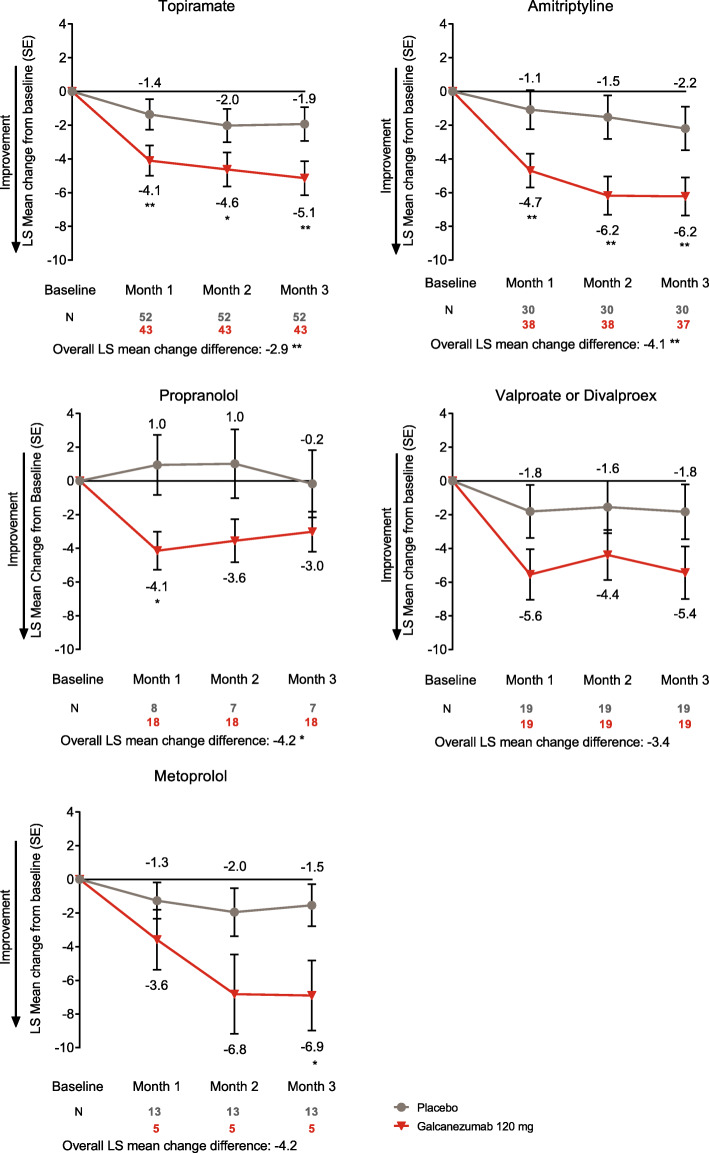
LS mean change from baseline in the number of monthly migraine headache days in patients who did not benefit from migraine preventive medication due to safety or tolerability. *p<0.05, **p<0.01 vs placebo. ^a^Only one patient discontinued onabotulinum toxin A due to safety/tolerability reasons; therefore, no statistical analyses were performed. Change from baseline showed a numerical reduction in monthly migraine headache days. Abbreviations: *LS* least-squares, *N* number of intent-to-treat patients, *SE* standard error

Galcanezumab-treated patients who did not tolerate topiramate, amitriptyline, or propranolol all had a significant reduction in overall mean monthly migraine headache days across months 1–3 compared to placebo (all *p* < 0.05; Fig. 2). Patients previously treated with valproate/divalproex or metoprolol experienced a larger numerical reduction in mean monthly migraine headache days with galcanezumab, but the reduction was not statistically significant, likely due to the small number of patients in these subgroups. Only one patient discontinued onabotulinum toxin A due to safety/tolerability reasons; therefore, no statistical analyses were performed. This patient was assigned to the galcanezumab group, had 8 monthly migraine headache days at baseline, and experienced an average reduction of 2 monthly migraine headache days over months 1–3.

## Discussion

Prescribing a migraine preventive medication that is efficacious and well-tolerated has the potential to increase adherence, reduce multiple medication switches, and ultimately improve patient outcomes [8–10, 12]. Many oral standard-of-care treatments are borrowed from other disease states, have suboptimal efficacy and poor tolerability, and require long titration periods [3–5]. Galcanezumab specifically targets the underlying mechanism of migraine, effectively reduces migraine headache frequency, is well-tolerated, and does not require titration or laboratory monitoring [20].

Galcanezumab was effective in reducing mean monthly migraine headache days in patients who had not previously benefited from topiramate, amitriptyline, propranolol, valproate or divalproex, onabotulinum toxin A, and metoprolol due to lack of efficacy or safety/tolerability. Efficacy was also demonstrated by a greater proportion of galcanezumab-treated patients with ≥ 50 % reduction in monthly migraine headache days compared to placebo. This threshold was used because it is widely considered to be clinically meaningful [21, 22].

Effect size tends to be larger when patients have failed multiple prior preventive medications. This is due to a low placebo response, likely because this patient population has lower expectations [18, 23, 24]. In this post hoc analysis, the effect size is largest in the group of patients who previously did not benefit from onabotulinum toxin A, most of whom experienced inadequate efficacy on this prior preventive. In this subgroup, patients treated with galcanezumab experienced 6.3 fewer mean monthly migraine headache days compared to 1.5 in placebo. These patients also achieved the largest 50 % response rate despite having the highest baseline monthly migraine headache days. This large effect size may be attributed to the greater number of prior preventive failures because patients often have to demonstrate inadequate response or intolerability to oral standard-of-care migraine preventive treatments in order to qualify for onabotulinum toxin A. These findings are consistent with a prior published post hoc analysis of patients treated with galcanezumab that had previous onabotulinum toxin A treatment failure [25], although the effect size in this analysis is even larger than previously reported.

The reduction in monthly migraine headache days with galcanezumab treatment was accompanied by an improvement in patients’ quality of life as measured by the MSQ-RFR, which assesses the effect of migraine on daily social and work-related activities [19]. The minimal important difference for group-level analyses of MSQ-RFR is 3.2 [26]. Patients treated with galcanezumab experienced an increase in their mean score by at least 10 points compared to patients in the placebo group, regardless of previous preventive medication failure. Prior studies have shown that despite taking standard-of-care preventive therapy, 29.2 % of patients with EM and 73.2 % of patients with CM have moderate-to-severe headache-related disability, indicating ongoing burden of disease despite treatment [8]. Improvement in quality of life, as seen in MSQ-RFR scores with galcanezumab, demonstrate the potential to help relieve this burden and improve patients’ lives.

The current American Headache Society guidelines state that patients should experience inadequate efficacy or intolerability of a six-week trial of at least two standard-of-care preventive treatments (and moderate disability in patients with 4–7 monthly headache days) before initiating a CGRP mAb [27]. Patients enrolled in this trial had an average of 3.5 prior preventive treatment failures in their lifetime and a baseline MSQ-RFR of approximately 45, which is indicative of poor quality of life. Starting galcanezumab earlier in a patient’s course of treatment could reduce monthly migraine headache days and improve quality of life sooner, leading to reduced disability and improved patient outcomes.

Limitations of this study include the post hoc nature of these analyses. The study was not powered for the analyses presented in this manuscript. Patients who had treatment failure of more than four standard-of-care migraine preventive medication categories in the past 10 years and patients with serious or unstable medical conditions such as serious cardiovascular conditions were excluded from the study. Enrolled patients were primarily female, Caucasian, and middle-aged, which may limit generalizability of the results. The effect of galcanezumab on patients who did not benefit from certain prior preventive treatments due to intolerability may be limited by the small sample size. Further, the three-month duration of these post hoc analyses may not be long enough to demonstrate the full effect of treatment. Nonetheless, consistency of efficacy across multiple outcome measures including migraine headache day reduction, response rates, and improvement in quality of life support the efficacy of galcanezumab in this difficult-to-treat patient population.

## Conclusions

In this population, galcanezumab 120 mg monthly (with a 240 mg loading dose) was effective for patients who previously did not benefit from topiramate, amitriptyline, propranolol, valproate or divalproex, onabotulinum toxin A, and/or metoprolol. This efficacy was consistent whether it was measured by reduction in monthly migraine headache days, ≥ 50 % response rates, or improvement in quality of life based on MSQ-RFR. This analysis supports the clinical value of galcanezumab in patients who have tried, but not benefited from commonly prescribed migraine preventive medications.

## Data Availability

Lilly provides access to all individual participant data collected during the trial, after anonymization, with the exception of pharmacokinetic or genetic data. Data are available to request 6 months after the indication studied has been approved in the United States and European Union and after primary publication acceptance, whichever is later. No expiration date of data requests is currently set once data are made available. Access is provided after a proposal has been approved by an independent review committee identified for this purpose and after receipt of a signed data sharing agreement. Data and documents, including the study protocol, statistical analysis plan, clinical study report, blank or annotated case report forms, will be provided in a secure data sharing environment. For details on submitting a request, see the instructions provided at www.vivli.org.

## Abbreviations

CGRP: Calcitonin gene-related peptide
CM: Chronic migraine
EM: Episodic migraine
GMB: Galcanezumab
LS: Least-squares
mAb: Monoclonal antibody
MSQ-RFR: Migraine-Specific Questionnaire Role Function-Restrictive
n: Number of patients within each specific category
N: Number of intent-to-treat patients
PBO: Placebo
SD: Standard deviation
SE: Standard error
